# How much of the female disadvantage in late-life cognition in India can be explained by education and gender inequality

**DOI:** 10.1038/s41598-022-09641-8

**Published:** 2022-04-05

**Authors:** Urvashi Jain, Marco Angrisani, Kenneth M. Langa, T. V. Sekher, Jinkook Lee

**Affiliations:** 1 Department of Economics, Finance and Real Estate, University of South Alabama, Mobile, USA; 2 Center for Economic and Social Research, University of Southern California, 635 Downey Way, Los Angeles, CA 90089-333 USA; 3 Department of Economics, University of Southern California, 635 Downey Way, Los Angeles, CA 90089-333 USA; 4 Department of Internal Medicine, University of Michigan Medical School, Ann Arbor, USA; 5 Institute for Social Research, University of Michigan, Ann Arbor, USA; 6 Veterans Affairs Ann Arbor Center for Clinical Management Research, Ann Arbor, MI USA; 7 International Institute for Population Sciences, Mumbai, India

**Keywords:** Neurology, Risk factors

## Abstract

In social environments characterized by high levels of gender inequality, women fare worse than men in human capital accumulation and health. We examine the association of gender inequality with female disadvantage in late-life cognitive function, using newly available data from Wave 1 (2017–2019) of the Longitudinal Aging Study in India (LASI), representative of the Indian population over the age of 45. We find a substantial female gap in cognition among mid-aged and older adults in India; early life socioeconomic conditions and education explain up to 74 percent of the female disadvantage in cognition, and model predictions suggest that it takes nine years of education on average to overcome this deficit. However, further contextualizing the environment, we find that the level of education at which differences in late-life cognition between women and men become negligible increases with the degree of gender inequality.

## Introduction

In environments characterized by high levels of gender inequality, women fare worse than men in human capital accumulation and health^1–3^. In this paper, we examine the female disadvantage in late-life cognitive functioning and its association with gender inequality. Prior studies have documented the existence of gender disparities in late-life cognition in India^4–6^, China^7,8^ and other developing countries^9,10^, but not in more developed economies, where older women perform no worse or even better than older men on cognitive testing^11,12^. The difference in educational attainment between men and women has been proposed as an important contributor to the observed gender gap in late-life cognition in developing countries, and as a possible explanation for why such a gap is negligible in developed countries where men and women have attained similar levels of education^13,14^. Educational attainment typically expands with economic development leading to a decrease in educational gender disparities^15^. For instance, Lei et al.^7^ found that educational expansion has benefited women more than men in China, contributing to a decreased gender gap in cognition in younger cohorts compared to older cohorts. India presents an interesting case study, as the female gap in late-life cognition remains largely unaccounted for even after controlling for education^16,17^.

In this study, we document the extent to which older Indian women are at a disadvantage in cognitive functioning compared to their male counterparts. We rely on a large and representative sample of the Indian population over the age of 45. We utilize geographic variation across India in gender inequality to examine the relationship between macro-level gender inequality and cognition, controlling for a wide range of individual-level characteristics. While previous research has found significant association between country-level gender inequality and female disadvantage in late-life physical health (as measured by the onset of limitations with Activities of Daily Living (ADL)^18^, most of the countries examined fare better than India in terms of gender equality. Moreover, cross-country analyses may mask within-country differences in gender inequality, which we are able to capture with our data collected across the entire Indian nation. Within India, significant cross-state variation in gender inequality has been documented, evidenced by heterogeneity in imbalanced sex ratios^19^, gender discrimination^20^, and in differences in educational attainment by gender across areas^5^. In this paper, we quantify gender differences in late-life cognition and relate them to the socio-cultural context faced by individuals, focusing explicitly on the degree of gender discrimination across states.

## Results

### Gender differences in education and late-life cognition

Table 1 presents the sample summary statistics for all the variables used in the analysis, separately for men and women. The most striking difference by gender is observed for educational attainment, with 62 percent of the women never having attended any school compared with 31 percent of sampled men. The fraction of women at old ages is slightly larger than that of men, reflecting longer life expectancy.

**Table 1:** Summary statistics.

	Women (N = 29,660)		Men (N = 26,048)		Difference
	Mean	SD	Mean	SD	
**Age**					
45–49	0.21	0.41	0.20	0.40	0.01***
50–54	0.17	0.38	0.17	0.37	0
55–59	0.16	0.37	0.15	0.35	0.01***
60–64	0.16	0.36	0.15	0.36	0
65–69	0.13	0.34	0.14	0.35	− 0.01***
70–74	0.08	0.27	0.09	0.29	− 0.01***
75–90	0.09	0.29	0.10	0.30	− 0.01***
**Levels of education**					
None	0.62	0.49	0.31	0.46	0.30***
Less than primary	0.10	0.29	0.13	0.34	− 0.04***
Primary completed	0.11	0.31	0.15	0.36	− 0.04***
Middle school	0.07	0.25	0.13	0.33	− 0.06***
Secondary school	0.06	0.23	0.12	0.33	− 0.07***
Higher secondary & above	0.06	0.23	0.15	0.36	− 0.09***
Father attended any school	0.27	0.44	0.29	0.45	− 0.02***
Height (cm)	150.01	6.47	162.33	6.89	− 12.33***
**Religion**					
Hindu	0.76	0.43	0.76	0.43	0
Muslim	0.09	0.29	0.09	0.29	0
Others	0.15	0.36	0.15	0.36	0
**Caste**					
General	0.27	0.44	0.27	0.44	0
Scheduled caste/scheduled tribe	0.33	0.47	0.33	0.47	0
Other backward castes	0.40	0.49	0.40	0.49	0
Rural	0.67	0.47	0.67	0.47	− 0.01*
**Region**					
North/east/northeast	0.43	0.49	0.41	0.49	0.02***
South/centre/west	0.57	0.49	0.59	0.49	− 0.02***

*Data source* Longitudinal Ageing Study in India (LASI), 2017–2019.

All statistics are weighted. ***p < 0.01, **p < 0.05, *p < 0.1.

**Figure 1:**
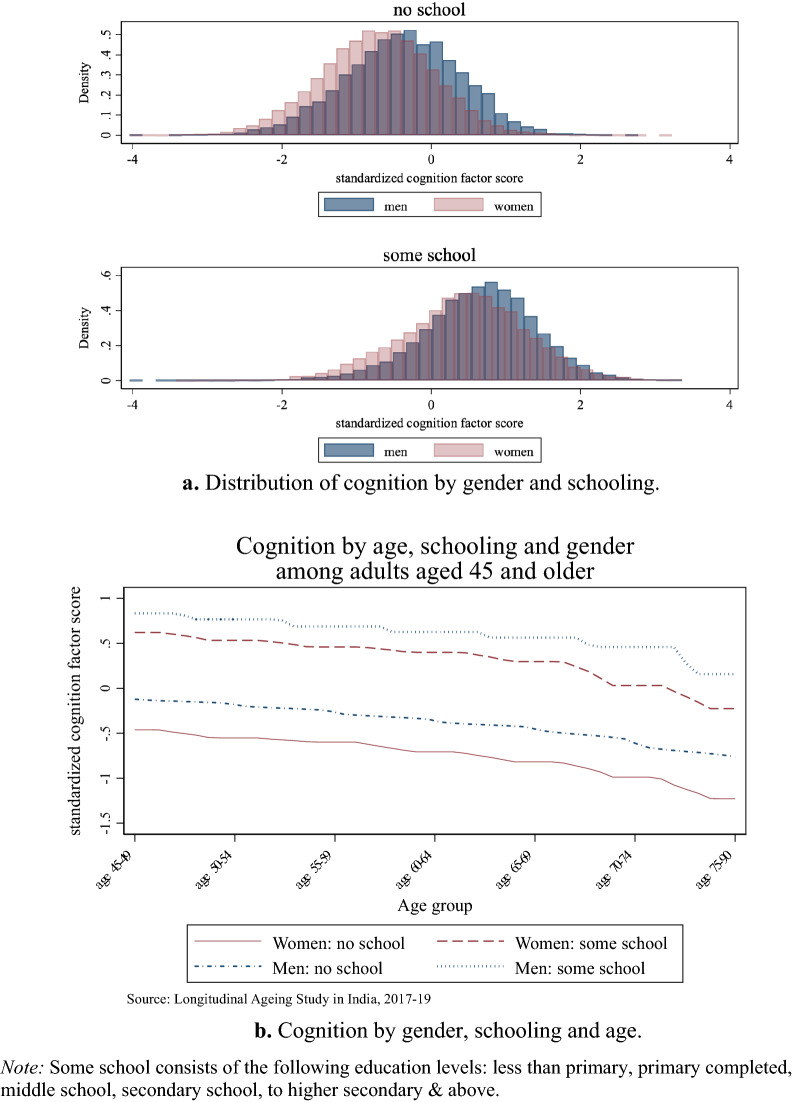
(**a**) Distribution of cognition by gender and schooling. (**b**) Cognition by gender, schooling and age.

In Fig. 1a, we present the distribution of a general cognitive factor score by gender, separately for individuals with no formal education and for those with some schooling. The score distribution for men is to the right of that for women, indicating a systematic female gap in cognitive function, which is more pronounced within the sub-sample of individuals who never attended school.

Figure 1b shows cognitive function stratified by gender, schooling, and age; while cognitive test score declines monotonically with age, gender differences persist across all age groups. Within the sub-sample of individuals with no formal education, the female gap in cognition becomes slightly wider after age 70. This may stem from differential mortality by innate ability and gender. Since men have lower life expectancy than women and higher ability individuals tend to live longer, in older cohorts surviving men would have higher ability than surviving women. Among individuals with some schooling, the female gap in late-life cognition is somewhat narrower within younger than older cohorts, plausibly reflecting a narrower female gap in educational attainment. Our descriptive analysis reveals substantial gender differences in late-life cognitive ability even after controlling for education and age. We therefore analyze the extent to which, in the Indian context, the observed female gap within groups is associated with the degree of gender discrimination faced by women in the place where they live.

### Gender inequality index and its association with gender difference in cognition at state level

**Figure 2:**
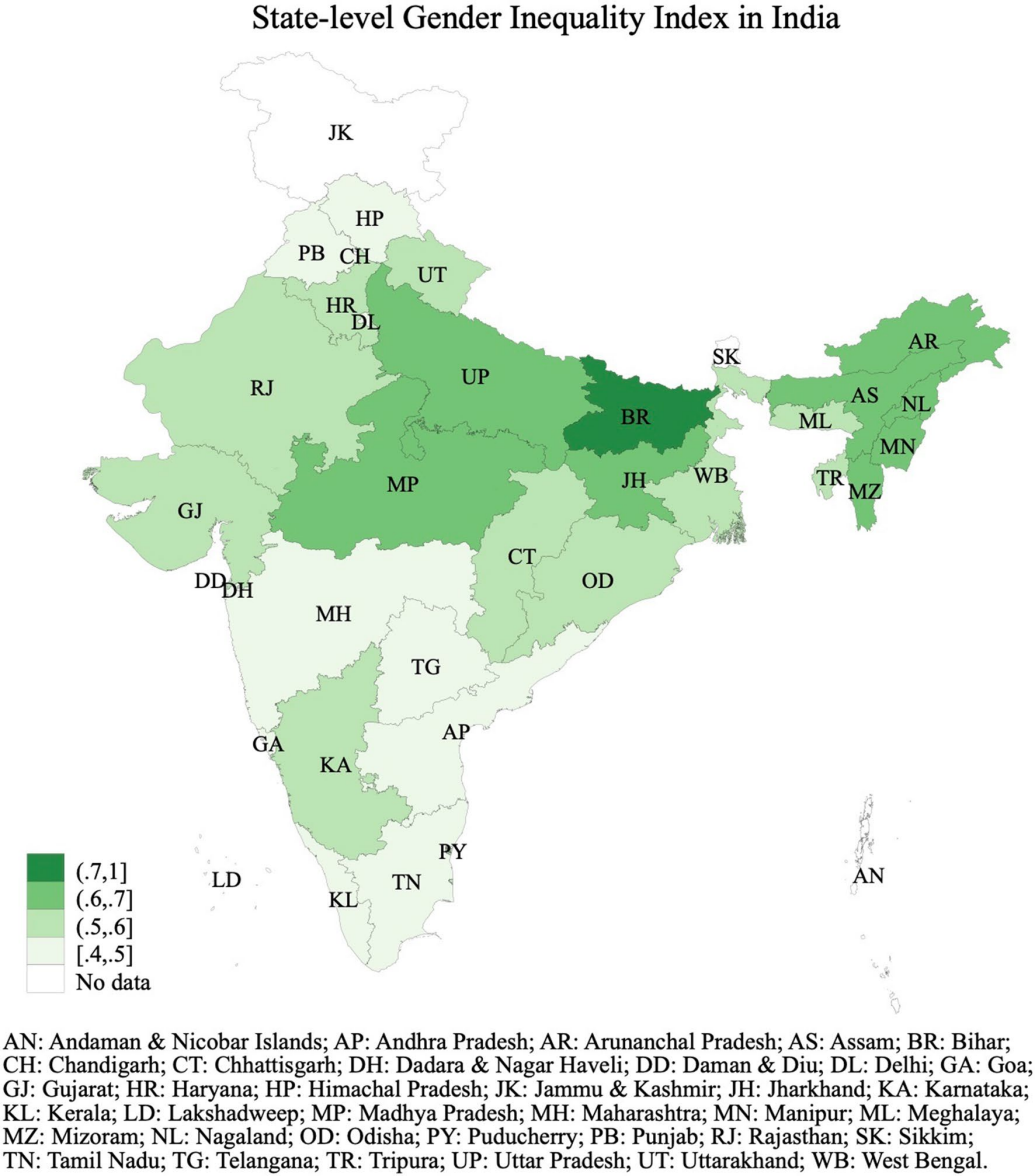
State-level Gender Inequality Index. Regions of India are defined as follows—North (Jammu and Kashmir, Himachal Pradesh, Punjab, Uttarakhand, Haryana, Delhi, Rajasthan, and Uttar Pradesh); East (Bihar, Jharkhand, West Bengal, Odisha); Northeast (Arunachal Pradesh, Nagaland, Manipur, Mizoram, Tripura, Meghalaya, Assam); Centre (Chhattisgarh, Madhya Pradesh); West (Gujarat, Maharashtra, Goa); South (Andhra Pradesh, Karnataka, Kerala, Tamil Nadu, Puducherry, Telangana). The map in figure was generated by the authors using Stata, version 17.0. (https://www.stata.com).

Figure 2 presents the state-level Gender Inequality Index (GII), a composite measure that quantifies inequalities women face in reproductive health, empowerment, and labor market^21^, (the state-level score for each component is also reported in Supplemental Table S1). The GII ranges from 0 to 1, with higher values indicating greater gender inequality. Across the states for which the GII can be computed, Himachal Pradesh and Kerala exhibit the lowest value (0.45), while Bihar the highest (0.73). To put these numbers in context, we notice that, using the exact same definition, the GII for Mexico and Sweden are 0.40 and 0.05, respectively^16^.

**Figure 3:**
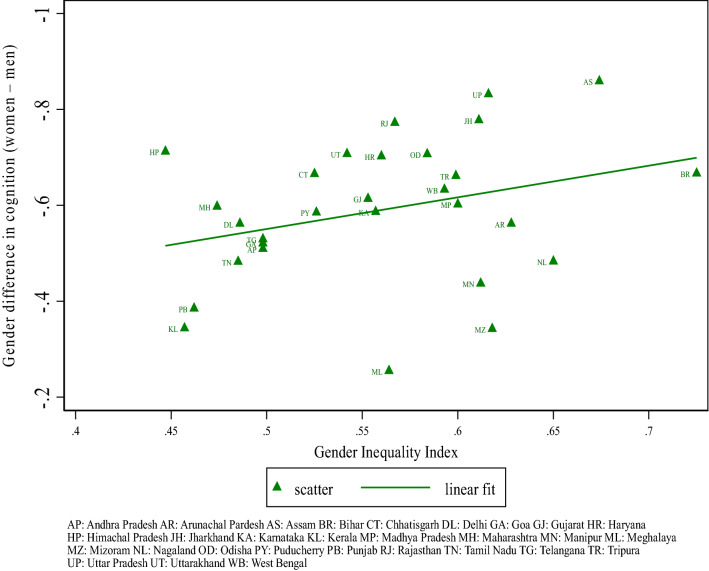
State-level gender inequality and average gender difference in cognition (age-adjusted).

Figure 3 shows the relationship between gender inequality across Indian states and gender difference in cognition (adjusted for age). It indicates that the female disadvantage worsens as the degree of gender inequality in the state of residence increases.

In Table 2, we provide a comprehensive set of men-women comparisons in the entire sample and by age, education, early life socio-economic background, religion, caste, and place of residence. Overall, women’s cognition factor score is significantly lower than men’s by 0.68 standard deviation. As shown already in Fig. 1b, the female gap persists across age groups, although it is lower among younger than older cohorts. Gender differences in cognitive health are highest among individuals with no schooling and decline with educational attainment, becoming statistically insignificant within the group with at least higher secondary education. There is also variation by region, with south, central, and western regions exhibiting substantially lower levels of gender difference compared to the north, east and northeast regions. Confirming the pattern in Fig. 3, we observe that the female disadvantage in late-life cognition is significantly more pronounced in states characterized by relatively higher levels of discrimination against women.

**Table 2:** Gender differences in cognition factor score among adults aged 45 and older in India.

	Women		Men		Difference
	Mean	SD	Mean	SD	
Full sample	− 0.37	0.96	0.31	0.92	− 0.68***
Age 45–49	0	0.93	0.58	0.85	− 0.58***
Age 50–54	− 0.15	0.95	0.44	0.86	− 0.59***
Age 55–59	− 0.26	0.9	0.34	0.88	− 0.60***
Age 60–64	− 0.39	0.9	0.24	0.91	− 0.63***
Age 65–69	− 0.55	0.89	0.13	0.92	− 0.68***
Age 70–74	− 0.78	0.86	− 0.03	0.95	− 0.75***
Age 75–90	− 1.08	0.9	− 0.29	0.97	− 0.79***
**Education level**					
No school	− 0.74	0.77	− 0.33	0.78	− 0.41***
Less than primary	− 0.2	0.75	0.14	0.75	− 0.34***
Primary completed	0.26	0.73	0.47	0.71	− 0.21***
Middle school	0.59	0.67	0.7	0.66	− 0.22***
Secondary school	0.87	0.66	0.92	0.64	− 0.05
Higher secondary & above	1.2	0.68	1.17	0.63	0.03**
**Father's education**					
No school	− 0.57	0.88	0.12	0.9	− 0.69***
Some school	0.24	0.96	0.81	0.8	− 0.57***
**Religion**					
Hindu	− 0.37	0.96	0.31	0.94	− 0.68***
Muslim	− 0.47	0.88	0.31	0.85	− 0.78***
Others	− 0.24	1.04	0.33	0.91	− 0.57***
**Caste**					
General	− 0.09	0.99	0.57	0.86	− 0.66***
Scheduled caste/scheduled tribe	− 0.67	0.87	0	0.92	− 0.67***
Other backward castes	− 0.35	0.95	0.35	0.91	− 0.70***
Urban	0.08	0.99	0.71	0.82	− 0.63***
Rural	− 0.59	0.87	0.12	0.91	− 0.71***
**Region**					
South/centre/west	− 0.29	1.01	0.33	0.96	− 0.62***
North/east/NE	− 0.45	0.91	0.29	0.89	− 0.74***
Lower gender inequality states^#^	− 0.25	1.01	0.36	0.93	− 0.61***
High gender inequality states^#^	− 0.49	0.9	0.28	0.88	− 0.77***

*Data source* Longitudinal Ageing Study in India (LASI), 2017–2019.

All statistics are weighted. ^#^Lower gender inequality states are those in the bottom quartile of the state-level gender inequality index (GII), high gender inequality states have GII in the top quartile. ***p < 0.01, **p < 0.05, *p < 0.1.

### Independent factors explaining gender differences in late-life cognition

It is apparent from Table 2 that several factors, which are plausibly correlated with each other, contribute to determine how older Indian women fare in terms of cognitive health compared to their male counterparts. To isolate the independent contribution of these factors to the observed female gap in late-life cognitive health, we rely on the multivariate regressions presented in Table 3, examining in particular the coefficient on female (in reference to male), with the full set of regression coefficients available in Supplemental Table S2. As can be seen in the first column of Table 3, conditional on age, the estimated female disadvantage in cognition compared to men amounts to 0.64 standard deviation of the cognition factor score.

**Table 3:** Multivariate regression estimates.

Variables	Model 1: gender and age	Model 2: add early-life SES, region	Model 3: add early-life nutrition	Model 4: add education	Model 5: add gender inequality
	(1)	(2)	(3)	(4)	(5)
	Dependent variable: cognition factor score				
**Gender (reference group: male)**					
Female	− 0.64*** (0.01)	− 0.63*** (0.01)	− 0.40*** (0.01)	− 0.17*** (0.01)	− 0.16*** (0.01)
**Region (reference group: south/west/centre)**					
North/east/northeast		− 0.06*** (0.01)	− 0.05*** (0.01)	− 0.03*** (0.01)	− 0.11*** (0.01)
Log (height)			2.88*** (0.08)	1.94*** (0.07)	2.03*** (0.07)
**Education level (reference group: no school)**					
Less than primary				0.45*** (0.01)	0.45*** (0.01)
Primary completed				0.76*** (0.01)	0.77*** (0.01)
Middle completed				0.95*** (0.01)	0.96*** (0.01)
Secondary school/matriculation				1.15*** (0.01)	1.16*** (0.01)
Higher secondary & above				1.35*** (0.01)	1.35*** (0.01)
Gender inequality index					0.94*** (0.05)
Observations	55,708	55,708	55,708	55,708	55,708
R-squared	0.19	0.34	0.35	0.51	0.51

*Data source* Longitudinal Ageing Study in India (LASI), 2017–2019.

All estimates are weighted. Covariates in Model 1 are gender and age groups. Additional covariates in Model 2 are caste (two groups indicating SC/ST/OBC or general), religion (three groups indicating Hindu, Muslim, or other religions), whether respondent’s father attended any school, residence in rural versus urban area, and region of residence in India (two groups indicating whether state of residence is in South/West/Centre or North/East/Northeast). Model 4 further controlled for logged value of respondent’s height. Model 5 adds individual’s highest education level, Model 6 adds state-level value of gender inequality index (GII). OLS estimates of coefficients on age, caste, religion, father’s schooling, rural residence, and the constant are not reported in this table but are available in supplementary material under Table S2. Standard errors in parentheses. ***p < 0.01, **p < 0.05, *p < 0.1.

The results in the second column of Table 3 reveal that, while significantly correlated with the average level of cognitive ability in the population, the indicators of caste, religion, father’s education, urbanicity, and region of residence do not explain the difference in cognition score between women and men. In contrast, adding the logarithm of height as a proxy for early-life nutrition (column 3 of Table 3) reduces the conditional female gap to 0.40, representing a nearly 38% reduction from the initial point estimate of 0.64. When we add the level of education to the set of explanatory variables (column 4 in Table 3), the estimated female gap decreases further to 0.17. Hence, educational attainment independently explains 36% of the difference in cognition score between women and men.

Finally, in the last column of Table 3, we augment the set of regressors with the state-level GII, which does not lead to a further reduction in the estimated female gap in cognitive ability to a considerable extent. In view of this finding, it is worth revisiting the positive relationship between the female disadvantage in cognitive health and the GII shown in Fig. 3. Such positive relationship plausibly stems from the fact that in states characterized by greater gender discrimination, women achieve lower levels of education, which in turn is associated with lower levels of cognitive functioning compared to men. Once educational attainment (as well as other demographics) is accounted for, there is no evidence that the difference between women’s and men’s cognition varies with the degree of gender inequality at the state level. We should note, however, that a state-level GII may mask heterogeneity across areas and poorly reflect the actual degree of gender discrimination experienced by individuals (we will return to this point in the discussion section). Overall, our model explains about three quarters of the observed female gap in cognition among older Indian adults. A quarter of the existing difference in late-life cognitive functioning between women and men remains unexplained and appears to be unrelated with the macro-level of gender discrimination as measured by our state-level GII.

### Gender differences in the protective effect of education on cognition

Given the prominent role played by education in shaping the cognition female gap, we explore whether the protective effect of education varies by gender. Hence, we estimate our richest model (the one in the last column of Table 3), adding an interaction term between gender and education, allowing us to compute the additional advantage, if any, that education might confer on women’s cognition compared to men (these estimates are available in Supplemental Table S3).

**Figure 4:**
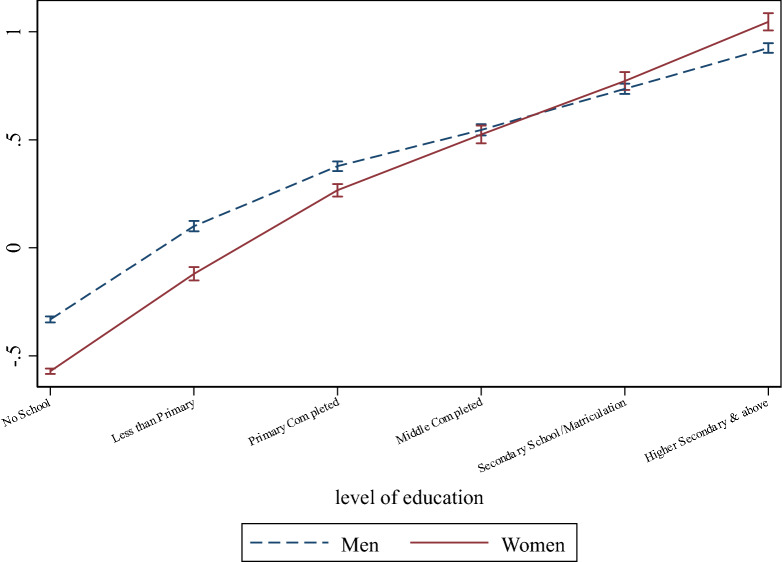
Predicted cognition scores by gender and education level. Predicted cognition scores with 95% confidence intervals are reported. Predictions are based on the estimates in the last column of Table S2. These estimates are adjusted for gender, age, caste, religion, rural/urban, region, father's education, height, education level, and interaction terms between gender and education levels, and derived from a regression using survey weights.

Figure 4 presents the cognition score predicted by these estimations at different levels of education, separately for women and men. For both genders, cognitive function increases monotonically with educational attainment. However, the marginal increase in cognition score for each additional level of education is generally larger for women than for men. Because of this pattern, the female gap at lower levels of education narrows progressively and becomes negligible in size and statistically indistinguishable from zero at middle school completion. At the highest level of education, our model predicts women’s cognition scores to be higher by 0.1 standard deviation compared to that of men. These findings plausibly stem from the role of education as a protective factor for late-life cognitive health and selectivity. Our empirical evidence suggests that, in the context of India, where women have been exposed to fewer cognitive stimuli than men through social and labor activities, it takes at least middle school for the female gap in late-life cognitive ability to be overcome. Precisely because inequality of opportunities by gender has been pervasive in India, it is likely that women who completed higher levels of education are a more selected group (e.g., they have higher ability, on average). This, in turn, may contribute to why the gap in cognitive score between women and men narrows as educational attainment increases. Both of these mechanisms are seemingly related to the degree of gender discrimination experienced by older Indian women.

**Figure 5:**
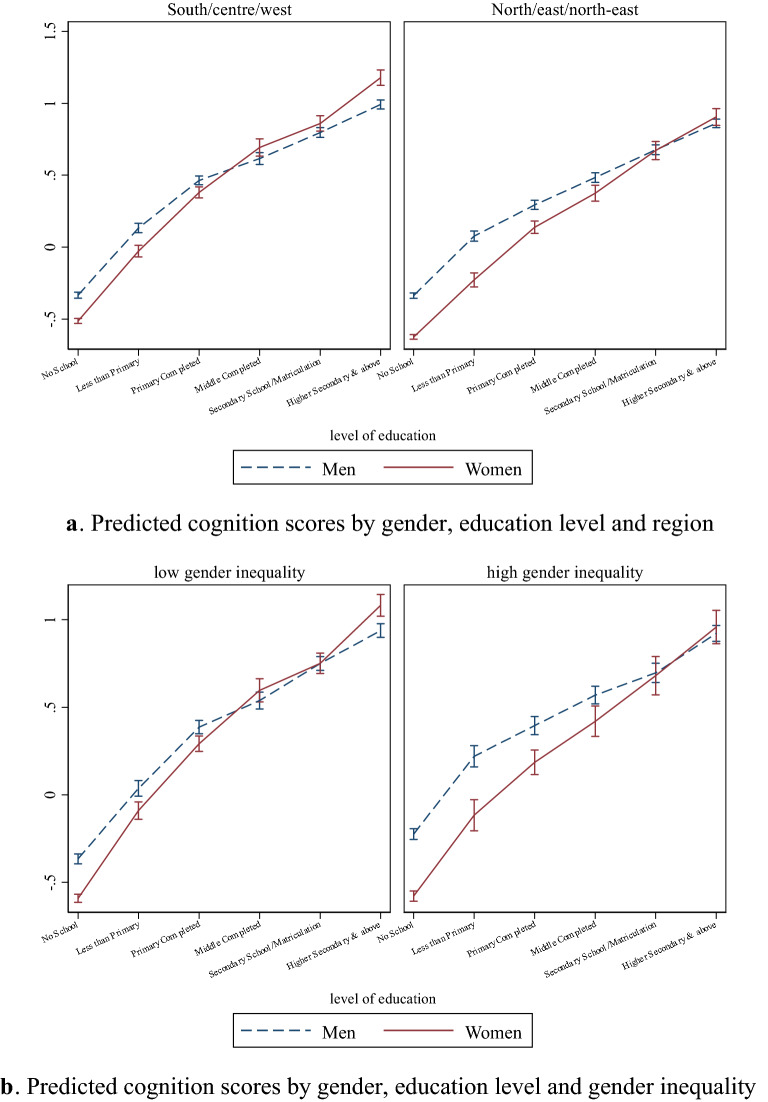
(**a**) Predicted cognition scores by gender, education level and region. (**b**) Predicted cognition scores by gender, education level and gender inequality. Predicted cognition scores with 95% confidence intervals are reported. Predictions are based on the estimates in Table S3. These estimates are adjusted for gender, age, caste, religion, rural/urban, region, father's education, height, education level, and derived from regressions using survey weights. The regression model used for (**a**) includes a triple-interaction between gender, education levels, and region. The regression model used for (**b**) includes a triple-interaction between gender, education levels, and the indicator for high versus low gender inequality states.

In view of this last observation, we investigate to what extent the differential protective effect of education by gender varies with the broad socio-cultural environment where individuals live. For this purpose, we estimate a model like the one in column 5 of Table 3 adding a triple interaction between gender, education, and an indicator for regions characterized by different degrees of gender-based discrimination (refer to Supplemental Table S3 for the full model). Specifically, we compare the South, Center, and West region, where gender inequality has been relatively less pronounced over the years, and the North, East, and Northeast region, where gender inequality has been historically more marked. We then predict cognition scores by gender at different levels of education, separately for these two regions. The results of this exercise are in Fig. 5a and reveal a very striking pattern (the results of a similar exercise using the triple interaction between gender, education and the urban/rural indicator are provided in Supplemental Figure S1). In the South, Center, and West region, the female gap in cognition score is negligible and not statistically significant at completion of primary school. After that, women fare systematically better than men in terms of cognitive functioning at each level of education. In contrast, within the North, East, and Northeast region, completion of secondary school is necessary before the female gap in cognition score becomes negligible. Moreover, there is no apparent female advantage at higher levels of education.

To better capture the degree of gender discrimination across areas, we perform an additional regression with the triple interaction between gender, education and a binary variable for whether a state is in the bottom or top quartile of the GII (see Supplemental Table S3 for the full model). In Fig. 5b we report the predicted level of cognition score from this model by gender at different levels of education, separately for these two groups of states. The observed patterns are remarkably similar to those observed in Fig. 3. Specifically, in states with low gender inequality, the gender gap in late-life cognitive health vanishes at the level of middle school. At higher secondary education and above, women perform significantly better than men. On the other hand, in the top quartile of the GII, a clear female disadvantage in cognition score is observed until secondary school. Importantly, women never exhibit an advantage compared to men even at higher levels of education. In summary, the level of education at which differences in late-life cognitive ability between women and men become negligible varies substantially with the socio-economic environment and the degree of gender discrimination faced by individuals.

## Discussion

Using data from India’s first nationally representative aging study, LASI (2017–2019), we find a significant gender gap in later-life cognition, which also exhibits substantial heterogeneity across the socio-cultural environments faced by individuals.

The most important contribution of this paper is the focus on how the interplay between gender, education, and contextual gender inequality shapes cognitive health at old ages. Specifically, we quantify whether and to what extent the protective effect of education on late-life cognition differs by gender and varies with the socio-cultural context where people live. We find that in environments of high gender inequality, women need more education in order to close the late-life cognition gap with men. We interpret this as evidence that, in areas characterized by greater gender discrimination, a higher cognitive reserve through education is necessary for women to compensate for receiving fewer cognitive stimuli through work and social activities. At the same time, barriers to accessing education in areas with greater gender discrimination create strong self-selection mechanisms. These imply that women who complete higher education tend to exhibit higher-than-average ability and motivation, which, in turn, are partly reflected in better cognitive tests performance at older ages. Our results confirm the importance of education as a protective factor for late-life cognitive functioning and suggest that education and macro-level gender equality play complementary roles in shaping cognitive health at older ages and determining differential outcomes for men and women. Our findings have important implications for not only India but also other developing countries.

Our study has a number of limitations. First, we construct and rely on a state-level measure of gender inequality, which is likely to mask important within-state differences in the degree of discrimination faced by women. This may be one of the reasons why our GII does not contribute to explain the observed gender gap in cognition. A GII at a finer geographic level that may better capture individuals’ exposure to gender inequality may exhibit a stronger predictive power. Second, we use cross-sectional data in our analysis and our estimates do not represent causal effects. Future research should exploit exogenous variation in education to infer the causal relationship between education and late-life cognition in India and document how it varies with the socio-cultural environment faced by individuals. It should also examine how changes in gender inequality over time are associated with longitudinal changes in the female gap in cognitive health.

## Methods

### Data and measures

We use individual-level data on cognition and other demographic variables from a harmonized version of LASI^22^, the first nationally representative survey of the health, economic, and social wellbeing of the Indian population age 45 and older. The LASI sample is also representative of each of the Indian States and Union Territories except Sikkim and includes an over-sample of individuals over the age of 65. Spouses were also interviewed, regardless of age. Our analysis sample focuses on individuals between the ages of 45 to 90.

The main dependent variable is cognitive function, as measured by a general cognitive factor score^22,23^. We use this measure since it is a broad, summary score, capturing multiple cognitive domains. Specifically, the general cognitive factor score was constructed based on a graded response item theory model^24^, using data from cognitive tests administered as a part of the LASI main interview, as well as a part of the Harmonized Diagnostic Assessment of Dementia for the LASI (LASI-DAD), an in-depth study of late-life cognition and dementia for a sub-sample of 4096 LASI participants^25^. The LASI-DAD protocol included a rich battery of neuropsychological tests (a total of 53 cognitive test items), including 11 overlapping tests with the main LASI, which allows leveraging information from the more intensive screening of LASI-DAD for the full LASI cohort. Conceptually, this approach estimates cognition as a latent trait based on a broad range of measured performance on tasks ranging from basic orientation (e.g., knowing the date) to more difficult tasks (e.g., naming as many animals as possible within 60 s). Prior to construction of this score, we imputed values for respondents with missing information^26^ using demographic and health variables. To assess the precision of our general cognitive function score, we evaluated model-estimated standard errors for each observation and flagged observations with poor marginal reliabilities. We observed that 97% of observations had marginal reliabilities above 70%, a level which is generally accepted for epidemiologic research^27^. Importantly, these derived cognitive scores were not sensitive to inclusion of items dependent on literacy (correlation between scores using literacy vs scores using non-literacy items was 0.995).

As we attempt to explain the gender difference in cognition, we use individual-level covariates measuring age, early-life socioeconomic status, childhood nutrition, and education. Gender is self-report by the respondents, with approximately 53% of the analysis sample being women. Age is also self-reported. We divide age into groups of five years from 45 to 49 to 70–74; ages 75 and above are grouped together due to the relatively small number of individuals in this age range. Early-life socioeconomic status is captured by caste (general compared with historically disadvantaged groups, namely Scheduled Caste, Scheduled Tribe and Other Backward Castes), religion (the majority group of Hindus compared with the largest minority group of Muslims, and a third category of all other minority religions), and father’s education (whether attended any school). Indicators for residence in rural versus urban area, and regions (states of India grouped into two: as North, East and Northeast versus West, Central and South) are also used as covariates to capture differences in socio-cultural and economic environments. Early-life nutrition is proxied for by height (measured by survey interviewers)^28^. Educational attainment is the highest level completed by the individual, indicated by five levels—no school, less than primary school, primary school completed, middle school, secondary school, and higher secondary and above.

Macro-level gender inequality is gauged by constructing a state-level GII. The GII was first conceptualized and calculated by the UNDP for 137 countries in 2010^29^. It is a composite measure which captures gender inequality across three domains—reproductive health, empowerment, and labor force participation—and does not allow high achievement in one dimension to compensate for low achievement in other dimension(s). It quantifies the loss of achievement due to (or opportunity cost of) gender inequality, with values between 0 to 1 (indicating 0 to 100% loss). The GII is a more concise measure of gender inequality compared to examining specific and multiple parameters of gender inequality. Since the aforementioned components of the GII index are available at the state-level, but not at a finer geographic or administrative level, we adopt a measure of gender inequality at the state level.

The first domain of GII, reproductive health, is captured by the maternal mortality ratio and adolescent fertility rates—these are available for the bigger states for 2011–2013 and 2012 respectively from the Sample Registration System (SRS) reports provided by the Census of India^30,31^. The second domain of GII, empowerment, is measured by the share of the parliamentary seats held by each gender, and proportion of population with at least secondary education by each sex. We construct the average share of state legislatures seats held by men and women during the period of from 1977 to 2015 using state-level data on parliamentary representation provided by Bhavnani^32^. We do not use a particular year since state elections are held every five years and are not synchronous across the country. State-level data on the proportion of population with at least secondary education by sex is available for all states for the year 2014 from the National Sample Survey Office^33^. State-level data on the third domain of the GII, labor force participation, is available for 2011–2012 from the Ministry of Statistics and Programme Implementation^34^. Out of a total of 35, we are able to construct the GII for 29 states and union territories of India using computation tools provided by the UNDP^35^. One state and five small union territories are excluded due to unavailable data on reproductive health and political representation. Overall, these excluded observations constitute less than 10% of the total sample.

### Statistical analysis

First, we document the magnitude of the gender disparity in cognition, testing for statistically significant differences among men and women in the entire sample, and among sub-groups by age, education, socioeconomic status, geographic region, and states with high versus low gender inequality.

We then quantify the extent to which all these factors help explain the observed gender difference in late-life cognition estimating multivariate regressions by Ordinary Least Squares (OLS). We begin with a linear regression model with cognition as the dependent variable, and binary indicators for female and age groups as covariates. In model 2, we include further covariates capturing region and socioeconomic status. In model 3, we add childhood nutrition (provided by height). Model 4 also controls for education. Model 5 controls for macro-level gender inequality (as captured by the GII). The main coefficient of interest in these regression models is the one of the female indicator, which measures the conditional female gap in late-life cognition. We examine whether and how this coefficient changes across specifications. In particular, we document the extent to which its magnitude decreases when more covariates are included in the model, as this reflects the ability of the additional regressors to explain the observed female gap.

Third, guided by the findings of these OLS regressions, we examine if education has a differential protective effect for late-life cognitive health for women and men. For this purpose, we estimate our richest regression model adding an interaction term between gender and education. This allows us to quantify to what extent each education level is differentially beneficial for women’s cognition compared to men’s. A by-product of this model is the identification of the education level, if it exists, at which the residual (after taking all the other demographic and contextual factors into account) female disadvantage in late-life cognitive health vanishes and becomes statistically indistinguishable from none.

Finally, we pose the question of whether the differential protective effect of education by gender varies with the context, especially the degree of gender-based inequality. To answer this question, we estimate our richest regression model with triple interaction between gender, education level and two different indicators capturing heterogeneity in socio-cultural context. The first is a binary variable for South, Center, and West region—characterized by relatively high gender inequality—versus the North, East, and Northeast region—which exhibits lower degrees of gender inequality; the second is a binary variable for whether the respondent’s states of residence is in the bottom or in the top quartile of the GII. This exercise informs us about whether the level of education at which the female gap in late-life cognitive health disappears varies depending on the socio-cultural environment and the degree of gender discrimination faced by individuals. Essentially, these estimates help us understand if a certain level of educational attainment has a different protective effect for women in different contexts and are therefore informative of potential complementarities between education and environmental circumstances in shaping women’s cognitive health in the long-term. All summary statistics and regression estimates employ survey weights which take into account the sampling design of LASI and adjust for differential non-response across demographic groups^22^.

## Supplementary Information

Supplementary Tables.
