# Supplementary material for: How much of the female disadvantage in late-life cognition in India can be explained by education and gender inequality
Source: Sci Rep. 2022 Apr 5;12:5684. doi: 10.1038/s41598-022-09641-8 (PMC8983756; doi:10.1038/s41598-022-09641-8)
Supplement: Supplementary file 1 — Supplementary Tables. [file 41598_2022_9641_MOESM1_ESM.docx]

| **Table S1.** Gender Inequality Index Across States | | | | | | | | | |
| --- | --- | --- | --- | --- | --- | --- | --- | --- | --- |
| State | Maternal mortality ratio (deaths per 100,000 live births) | Adolescent fertility ratio (births per 1000 women ages 15-19) | Seats in parliament held by (% of total) | | Population with at least secondary education (% ages 25 and older) | | Labour force participation rate (%) | | Gender Inequality Index |
|  |  |  | Female | Male | Female | Male | Female | Male |  |
| Andhra Pradesh | 92 | 36.8 | 6.1 | 93.9 | 18.4 | 27.9 | 36.1 | 60 | 0.498 |
| Arunachal Pradesh | 300 | 49.8 | 3.3 | 96.7 | 19.4 | 27.8 | 25.6 | 48.8 | 0.628 |
| Assam | 300 | 49.8 | 4.3 | 95.7 | 18.4 | 23.6 | 12.6 | 56.5 | 0.674 |
| Bihar | 208 | 37.7 | 3.0 | 97.0 | 10.3 | 19.5 | 5.7 | 48.2 | 0.725 |
| Chhattisgarh | 221 | 31.1 | 8.3 | 91.7 | 12.5 | 22.3 | 38.2 | 55.3 | 0.525 |
| Goa | 68 | 26.3 | 5.8 | 94.2 | 34.3 | 42.8 | 19.3 | 55.6 | 0.498 |
| Gujarat | 112 | 27.6 | 4.2 | 95.8 | 20.7 | 29.6 | 22.2 | 60.4 | 0.553 |
| Haryana | 127 | 17.9 | 4.2 | 95.8 | 25.8 | 35.6 | 14.5 | 53.3 | 0.560 |
| Himachal Pradesh | 141 | 16.5 | 5.0 | 95.0 | 35.2 | 45.6 | 49.8 | 55.5 | 0.447 |
| Jharkhand | 208 | 46.5 | 6.8 | 93.2 | 13.1 | 22.1 | 17.6 | 53.3 | 0.611 |
| Karnataka | 133 | 33.8 | 4.8 | 95.2 | 24.8 | 33.6 | 24.6 | 61 | 0.557 |
| Kerala | 61 | 21.3 | 4.7 | 95.3 | 37.4 | 38.5 | 24.8 | 57.9 | 0.457 |
| Madhya Pradesh | 221 | 34.2 | 5.2 | 94.8 | 13.4 | 22.5 | 20.8 | 55.6 | 0.600 |
| Maharashtra | 68 | 26.3 | 4.9 | 95.1 | 28 | 36.5 | 29 | 57.2 | 0.474 |
| Manipur | 300 | 49.8 | 4.6 | 95.4 | 33.6 | 44.2 | 25.2 | 51.2 | 0.612 |
| Meghalaya | 300 | 49.8 | 6.5 | 93.5 | 18.9 | 20.6 | 35.3 | 52.7 | 0.564 |
| Mizoram | 300 | 49.8 | 2.9 | 97.1 | 20.4 | 25.4 | 33.6 | 55.5 | 0.618 |
| Nagaland | 300 | 49.8 | 1.5 | 98.5 | 40.5 | 46.8 | 32.1 | 56.1 | 0.650 |
| Delhi | 104 | 8.4 | 7.4 | 92.6 | 45.8 | 48.7 | 11.1 | 54.8 | 0.486 |
| Odisha | 222 | 35.1 | 6.0 | 94.0 | 14.8 | 21.9 | 23.8 | 60.5 | 0.584 |
| Puducherry | 79 | 21 | 3.2 | 96.8 | 36.2 | 40.4 | 18 | 54.8 | 0.526 |
| Punjab | 141 | 7.5 | 5.5 | 94.5 | 29.8 | 35.7 | 20.3 | 58.1 | 0.462 |
| Rajasthan | 244 | 27.8 | 4.7 | 95.3 | 12.9 | 23.5 | 30.1 | 50.1 | 0.567 |
| Tamil Nadu | 79 | 21 | 3.7 | 96.3 | 26.6 | 34.5 | 30.8 | 60.4 | 0.485 |
| Telangana | 92 | 36.8 | 6.1 | 93.9 | 18.4 | 27.9 | 36.1 | 60 | 0.498 |
| Tripura | 300 | 49.8 | 8.0 | 92.0 | 10.1 | 17 | 28.2 | 59.8 | 0.599 |
| Uttar Pradesh | 285 | 26.2 | 4.1 | 95.9 | 16.5 | 24.1 | 16.3 | 50.4 | 0.616 |
| Uttarakhand | 285 | 26.2 | 7.6 | 92.4 | 26.9 | 39.4 | 26.3 | 47.9 | 0.542 |
| West Bengal | 113 | 59 | 5.2 | 94.8 | 18 | 22.9 | 19.2 | 61 | 0.593 |
| *Data sources*: Sample Registration System (SRS) reports 2012 & 2018, Census of India. India National and State Election Dataset, 2014. "Education in India", National Sample Survey Office Report No. 575. "Participation in the Economy", In: Women & Men in India 2017- A Statistical Profile. Social Statistics Division, Central Statistics Office, Ministry of Statistics and Programme Implementation, Government of India; 2018. Authors' calculations using "Calculating the Indices, Human Development Reports" by UNDP. | | | | | | | | | |

| **Table S2.** Multivariate regression estimates | | | | | |
| --- | --- | --- | --- | --- | --- |
|  | Model 1: gender and age | Model 2: add early-life SES, region | Model 3: add early-life nutrition | Model 4: add education | Model 5: add gender inequality |
|  | (1) | (2) | (3) | (4) | (5) |
| VARIABLES | *Dependent variable: Cognition factor score* | | | | |
|  |  |  |  |  |  |
| Female | -0.64*** | -0.63*** | -0.40*** | -0.17*** | -0.16*** |
|  | (0.01) | (0.01) | (0.01) | (0.01) | (0.01) |
| *Age (reference group: 45-49)* | | | | | |
| Age 50-54 | -0.14*** | -0.12*** | -0.11*** | -0.07*** | -0.07*** |
|  | (0.01) | (0.01) | (0.01) | (0.01) | (0.01) |
| Age 55-59 | -0.24*** | -0.20*** | -0.18*** | -0.11*** | -0.11*** |
|  | (0.01) | (0.01) | (0.01) | (0.01) | (0.01) |
| Age 60-64 | -0.36*** | -0.29*** | -0.26*** | -0.18*** | -0.18*** |
|  | (0.01) | (0.01) | (0.01) | (0.01) | (0.01) |
| Age 65-69 | -0.49*** | -0.42*** | -0.37*** | -0.27*** | -0.27*** |
|  | (0.01) | (0.01) | (0.01) | (0.01) | (0.01) |
| Age 70-74 | -0.69*** | -0.61*** | -0.55*** | -0.41*** | -0.41*** |
|  | (0.02) | (0.01) | (0.01) | (0.01) | (0.01) |
| Age 75-90 | -0.97*** | -0.88*** | -0.79*** | -0.62*** | -0.61*** |
|  | (0.02) | (0.01) | (0.01) | (0.01) | (0.01) |
| *Caste (reference group: general)* | | | | | |
| SC/ST/OBC |  | -0.20*** | -0.18*** | -0.01* | -0.02*** |
|  |  | (0.01) | (0.01) | (0.01) | (0.01) |
| *Religion (reference group: Hindu)* | | | | | |
| Muslim |  | -0.12*** | -0.12*** | 0.04*** | 0.03*** |
|  |  | (0.01) | (0.01) | (0.01) | (0.01) |
| Other |  | 0.06*** | 0.03** | -0.01 | 0.05*** |
|  |  | (0.01) | (0.01) | (0.01) | (0.01) |
| Father attended school |  | 0.53*** | 0.52*** | 0.11*** | 0.11*** |
|  |  | (0.01) | (0.01) | (0.01) | (0.01) |
| Rural |  | -0.48*** | -0.47*** | -0.22*** | -0.23*** |
|  |  | (0.01) | (0.01) | (0.01) | (0.01) |
| *Region (reference group: South, west, centre)* | | | | | |
| North, East, Northeast |  | -0.06*** | -0.05*** | -0.03*** | -0.11*** |
|  |  | (0.01) | (0.01) | (0.01) | (0.01) |
| Log (height) |  |  | 2.88*** | 1.94*** | 2.03*** |
|  |  |  | (0.08) | (0.07) | (0.07) |
| *Education level (reference group: no school)* | | | | | |
| Less than Primary |  |  |  | 0.45*** | 0.45*** |
|  |  |  |  | (0.01) | (0.01) |
| Primary Completed |  |  |  | 0.76*** | 0.77*** |
|  |  |  |  | (0.01) | (0.01) |
| Middle Completed |  |  |  | 0.95*** | 0.96*** |
|  |  |  |  | (0.01) | (0.01) |
| Secondary School/Matriculation |  |  |  | 1.15*** | 1.16*** |
|  |  |  |  | (0.01) | (0.01) |
| Higher Secondary & above |  |  |  | 1.35*** | 1.35*** |
|  |  |  |  | (0.01) | (0.01) |
| Gender Inequality Index |  |  |  |  | 0.94*** |
|  |  |  |  |  | (0.05) |
| Constant | 0.60*** | 0.93*** | -13.76*** | -9.84*** | -10.77*** |
|  | (0.01) | (0.01) | (0.42) | (0.37) | (0.37) |
|  |  |  |  |  |  |
| Observations | 55,708 | 55,708 | 55,708 | 55,708 | 55,708 |
| R-squared | 0.19 | 0.34 | 0.35 | 0.51 | 0.51 |
| Standard errors in parentheses. *** p<0.01, ** p<0.05, * p<0.1. All estimates are weighted. | | | | | |

| **Table S3. Regression Estimated for the Predictions** | | | |
| --- | --- | --- | --- |
|  | Regression estimated used for predictions in: | | |
|  | Figure 4 | Figure 5a | Figure 5b |
| VARIABLES |  | *Cognition score* | |
|  |  |  |  |
| Female | -0.24*** | -0.179*** | -0.226*** |
|  | (0.01) | (0.014) | (0.017) |
| *Education level (reference group: no school)* | | | |
| Less than Primary | 0.43*** | 0.467*** | 0.403*** |
|  | (0.01) | (0.019) | (0.025) |
| Primary Completed | 0.71*** | 0.798*** | 0.753*** |
|  | (0.01) | (0.018) | (0.022) |
| Middle Completed | 0.88*** | 0.949*** | 0.904*** |
|  | (0.01) | (0.023) | (0.027) |
| Secondary School/Matriculation | 1.07*** | 1.130*** | 1.116*** |
|  | (0.01) | (0.020) | (0.023) |
| Higher Secondary & above | 1.26*** | 1.325*** | 1.304*** |
|  | (0.01) | (0.019) | (0.023) |
| Female X Less than Primary | 0.02 | 0.018 | 0.099*** |
|  | (0.02) | (0.028) | (0.036) |
| Female X Primary Completed | 0.13*** | 0.095*** | 0.131*** |
|  | (0.02) | (0.027) | (0.032) |
| Female X Middle Completed | 0.22*** | 0.256*** | 0.284*** |
|  | (0.03) | (0.038) | (0.043) |
| Female X Secondary School/Matriculation | 0.28*** | 0.242*** | 0.226*** |
|  | (0.03) | (0.034) | (0.037) |
| Female X Higher Secondary & above | 0.36*** | 0.365*** | 0.370*** |
|  | (0.02) | (0.033) | (0.039) |
| Region: North/East/NE |  | -0.003 |  |
|  |  | (0.014) |  |
| Female X Region: North/East/NE |  | -0.109*** |  |
|  |  | (0.017) |  |
| Region: North/East/NE X Less than Primary |  | -0.053* |  |
|  |  | (0.027) |  |
| Region: North/East/NE X Primary Completed |  | -0.167*** |  |
|  |  | (0.026) |  |
| Region: North/East/NE X Middle Completed |  | -0.129*** |  |
|  |  | (0.029) |  |
| Region: North/East/NE X Secondary School/Matriculation |  | -0.117*** |  |
|  |  | (0.027) |  |
| Region: North/East/NE X Higher Secondary & above |  | -0.128*** |  |
|  |  | (0.025) |  |
| Female X Less than Primary X Region: North/East/NE |  | -0.035 |  |
|  |  | (0.043) |  |
| Female X Primary Completed X Region: North/East/NE |  | 0.037 |  |
|  |  | (0.040) |  |
| Female X Middle Completed X Region: North/East/NE |  | -0.077 |  |
|  |  | (0.051) |  |
| Female X Secondary School/Matriculation X Region: North/East/NE | | 0.040 |  |
|  |  | (0.051) |  |
| Female X Higher Secondary & above X Region: North/East/NE |  | -0.032 |  |
|  |  | (0.047) |  |
| GII top quartile |  |  | 0.141*** |
|  |  |  | (0.023) |
| Female X GII top quartile |  |  | -0.128*** |
|  |  |  | (0.022) |
| GII top quartile X Less than Primary |  |  | 0.042 |
|  |  |  | (0.040) |
| GII top quartile X Primary Completed |  |  | -0.132*** |
|  |  |  | (0.035) |
| GII top quartile X Middle Completed |  |  | -0.110*** |
|  |  |  | (0.038) |
| GII top quartile X Secondary School/Matriculation |  |  | -0.195*** |
|  |  |  | (0.037) |
| GII top quartile X Higher Secondary & above |  |  | -0.157*** |
|  |  |  | (0.032) |
| Female X Less than Primary X GII top quartile |  |  | -0.081 |
|  |  |  | (0.066) |
| Female X Primary Completed X GII top quartile |  |  | 0.014 |
|  |  |  | (0.056) |
| Female X Middle Completed X GII top quartile |  |  | -0.080 |
|  |  |  | (0.068) |
| Female X Secondary School/Matriculation X GII top quartile |  |  | 0.112 |
|  |  |  | (0.073) |
| Female X Higher Secondary & above X GII top quartile |  |  | 0.021 |
|  |  |  | (0.066) |
|  |  |  |  |
| Observations | 55,708 | 55,708 | 31,708 |
| R-squared | 0.52 | 0.518 | 0.511 |
| Standard errors in parentheses. *** p<0.01, ** p<0.05, * p<0.1. All estimates are weighted. Note: covariates included but not shown are the same as in Table S2. | | | |

**Figure S1.** Predicted cognition scores by gender and urbanicity

*Note:* Predictions derived from a regression of cognition score on the same explanatory variables as in Table S2 and a triple-interaction between gender, education levels, and urban/rural indicator.
